# Supplementary material for: Content-rich biological network constructed by mining PubMed abstracts
Source: BMC Bioinformatics. 2004 Oct 8;5:147. doi: 10.1186/1471-2105-5-147 (PMC528731; doi:10.1186/1471-2105-5-147)
Supplement: Additional File 2 — The original results of the above study (non-essential files are deleted to keep the file size under the limit set by BMC bioinformatics). [file 1471-2105-5-147-S2.bz2 › chilibotAdditionalFile2/dip05/311ID8405064E1360/html/TNFSF9_TNFRSF9.html]

 


 **TNFSF9** and **TNFRSF9** 
  
Found 93 abstracts in PubMed, retrieved 05.  
 

 What does Google say? 
 PDF only 
| .edu only 

---

**Interactive relationship** (e.g. stimulation, inhibition, etc)

**Stimulatory relationship**- 4 1BB  [ **TNFRSF9** ]  is a member of the tumor necrosis factor  [ **TNFSF9** ]  receptor superfamily and provides a costimulatory signal by interaction with 4 1BB  [ **TNFRSF9** ]  ligand  [ **TNFSF9** ]  expressed on antigen presenting cells.  Ref: 12706838 FEBS Lett, 2003
- An increase in the caspase activities was also observed in the HeLaTR cells transfected with 4 1BB  [ **TNFRSF9** ]  cDNA, and the 4 1BB  [ **TNFRSF9** ]  Ligand  [ **TNFSF9** ]  further increased the caspase activities of the HeLaTR cells overexpressing the 4 1BB  [ **TNFRSF9** ] .  Ref: 12846840 Eur J Biochem< MedlineTA>Eur J Biochem, 2003
**Neutral relationship**- Blocking 4 1BB  [ **TNFRSF9** ]  4 1BB  [ **TNFRSF9** ]  Ligand  [ **TNFSF9** ]  Interactions Prevents Herpetic Stromal Keratitis.  Ref: 12847221 J Immunol, 2003
- We used the murine model of HSK to demonstrate the importance of the interaction between an inducible T cell costimulatory receptor, 4 1BB  [ **TNFRSF9** ] , and its ligand, 4 1BB  [ **TNFRSF9** ]  ligand  [ **TNFSF9** ]  4 1BBL  [ **TNFSF9** ] , in the development of this disease.  Ref: 12847221 J Immunol, 2003
- In BALB c mice, HSK ordinarily induced by infection with the RE strain of herpes was prevented by blocking 4 1BB  [ **TNFRSF9** ]  4 1BBL  [ **TNFSF9** ]  interaction, either by deleting 4 1BB  [ **TNFRSF9** ]  in mutant 4 1BB  [ **TNFRSF9** ]  mice or by introducing mAbs against 4 1BBL  [ **TNFSF9** ] .  Ref: 12847221 J Immunol, 2003

**Non-interactive relationship** (e.g. studied together, co-existance, homology, etc.)

- Although treatment of the HeLaTR cells with T3 did not induce expression of NF kappaB reporter luciferase, even in the presence of the 4 1BB  [ **TNFRSF9** ]  Ligand  [ **TNFSF9** ] , it increased the caspase activities.  Ref: 12846840 Eur J Biochem< MedlineTA>Eur J Biochem, 2003
- Polyclonal CD4 T lymphocytes can be activated and expanded using anti CD3 and anti CD28 antibodies presented on magnetic beads, and CD8 T lymphocytes can be successfully expanded using a novel genetically engineered cell based technology that presents anti CD3 and anti CD28 along with the costimulatory molecule CD137  [ **TNFRSF9** ]  4 1BBL  [ **TNFSF9** ] .  Ref: 12857980 Immunol Res, 2003
